# Sevoflurane Induces Learning and Memory Impairment in Young Mice Through a Reduction in Neuronal Glucose Transporter 3

**DOI:** 10.1007/s10571-019-00779-0

**Published:** 2019-12-28

**Authors:** Jinpiao Zhu, Zongze Zhang, Junke Jia, Lirong Wang, Qiuyue Yang, Yanlin Wang, Chang Chen

**Affiliations:** Department of Anaesthesiology, Zhongnan Hospital, Wuhan University, East Lake Road, Wuhan, 430071 Hubei China

**Keywords:** Sevoflurane, GLUT3, Glucose metabolism, Apoptosis, Cognition

## Abstract

Sevoflurane, which is widely used in paediatric anaesthesia, induces neural apoptosis in the developing brain and cognitive impairment in young mammals. Glucose hypometabolism is the key pathophysiological modulator of cognitive dysfunction. However, the effects and mechanism of sevoflurane on cerebral glucose metabolism after its use as an anaesthetic and its complete elimination are still unknown. We therefore investigated the influence of sevoflurane on neuronal glucose transporter isoform 3 (GLUT3) expression, glucose metabolism and apoptosis in vivo and in vitro and on neurocognitive function in young mice 24 h after the third exposure to sevoflurane. Postnatal day 14 (P14) mice and neural cells were exposed to 3% sevoflurane 2 h daily for three days. We found that sevoflurane anaesthesia decreased GLUT3 gene and protein expression in the hippocampus and temporal lobe, consistent with a decrease in glucose metabolism in the hippocampus and temporal lobe observed by [18F] fluorodeoxyglucose positron emission tomography (18F-FDG PET). Moreover, sevoflurane anaesthesia increased the number of TUNEL-positive cells and the levels of Bax, cleaved caspase 3 and cleaved PARP and reduced Bcl-2 levels in the hippocampus and temporal lobe. Young mice exposed to sevoflurane multiple times also showed learning and memory impairment. In addition, sevoflurane inhibited GLUT3 expression in primary hippocampal neurons and PC12 cells. GLUT3 overexpression in cultured neurons ameliorated the sevoflurane-induced decrease in glucose utilization and increase in the apoptosis rate. These data indicate that GLUT3 deficiency may contribute to sevoflurane-induced learning and memory deficits in young mice.

## Introduction

Several clinical studies have examined the risk of anaesthetic neurotoxicity in childhood (DiMaggio et al. 2009; Wilder et al. 2009). Anaesthesia has also been suggested to be detrimental to cognitive development in young rodents and nonhuman primates(Li et al. 2007; Satomoto et al. 2009; Kodama et al. 2011; Paule et al. 2011; Jia et al. 2016; Vutskits and Davidson, 2017). Sevoflurane is one of the most frequently used volatile anaesthetics in infants and children because of its rapid induction and recovery times and because it causes less irritation to the airway than other anaesthetics (Wallin et al. 1975; Sarner et al. 1995). However, the underlying mechanisms by which sevoflurane delays neurotoxicity remain unclear.

Sevoflurane not only suppresses brain activity but also decreases cerebral glucose metabolism during anaesthesia (Lenz et al. 1998; Schlunzen et al. 2010). However, whether sevoflurane decreases cerebral glucose utilization, especially in the developing brain, after its rapid elimination remains unclear. Glucose metabolism provides the fuel for physiological brain functioning and is the foundation of neuronal and non-neuronal cellular maintenance through the generation of ATP (Mergenthaler et al. 2013). In addition, glucose metabolism is a central player in programmed cell death pathways (King and Gottlieb 2009; Mergenthaler et al. 2013). Anaesthetic-induced caspase activation and neuroapoptosis depend on the age of the neurons (Kodama et al. 2011; Creeley et al. 2014; Deng et al. 2014). During the early stage of brain development, disturbed glucose metabolism in the brain may underlie the effects of sevoflurane-induced neurotoxicity on cognitive function.

Studies performed by Lenz et al. demonstrated a 66% decrease in mean cerebral glucose utilization at one minimum alveolar concentration (1 MAC) and a 41% decrease at 2 MAC during sevoflurane anaesthesia in rats (Lenz et al. 1998). The expression levels of glucose transporters are regulated in concert with metabolic demand and regional rates of cerebral glucose utilization (Simpson et al. 2007). GLUT1 is primarily responsible for the transport of glucose across the blood–brain barrier (BBB) (Gomez et al. 2010); GLUT3 is a neuronal glucose transporter that takes up the bulk of the glucose transported into neurons to maintain essential neural activities and viability (Simpson et al. 2008). In the brain, these two types of transporters have different functions, affinities, capacities, and tissue distributions. However, whether sevoflurane reduces brain glucose metabolism by regulating neural glucose transporters after anaesthesia is unclear.

The goal of the present research was to determine whether sevoflurane-induced glucose hypometabolism is involved in cognitive impairment in the young after anaesthesia. In the present study, we assessed cerebral glucose metabolism and cognitive function in young mice 24 h after the third exposure to sevoflurane. We also observed changes in the levels of the glucose transporter GLUT3 and neural apoptosis in specific brain regions. To further elucidate whether GLUT3 contributes to sevoflurane-induced glucose hypometabolism and neural apoptosis, primary hippocampal neurons and PC12 cells were transfected with lentivirus to induce the overexpression of GLUT3.

## Materials and Methods

### Animals

All protocols involving animals were approved by the Animal Ethics Committee of Zhongnan Hospital, Wuhan University, China (NO: SY02518071), and all animals were treated in accordance with the Guide for the Care and Use of Laboratory Animals from the National Institute of Health (Bethesda, Maryland, USA). The animal experiments were performed in the Animal Experiment Center of Zhongnan Hospital of Wuhan University. All efforts were made to minimize the number of animals used and their suffering. The mice were housed at a constant temperature of 23 ± 3 °C and a humidity of approximately 30% under a 12-h light/dark cycle (light from 7:00 to 19:00) with ad libitum access to food and water.

### Animal Anaesthesia

**Fig. 1:**
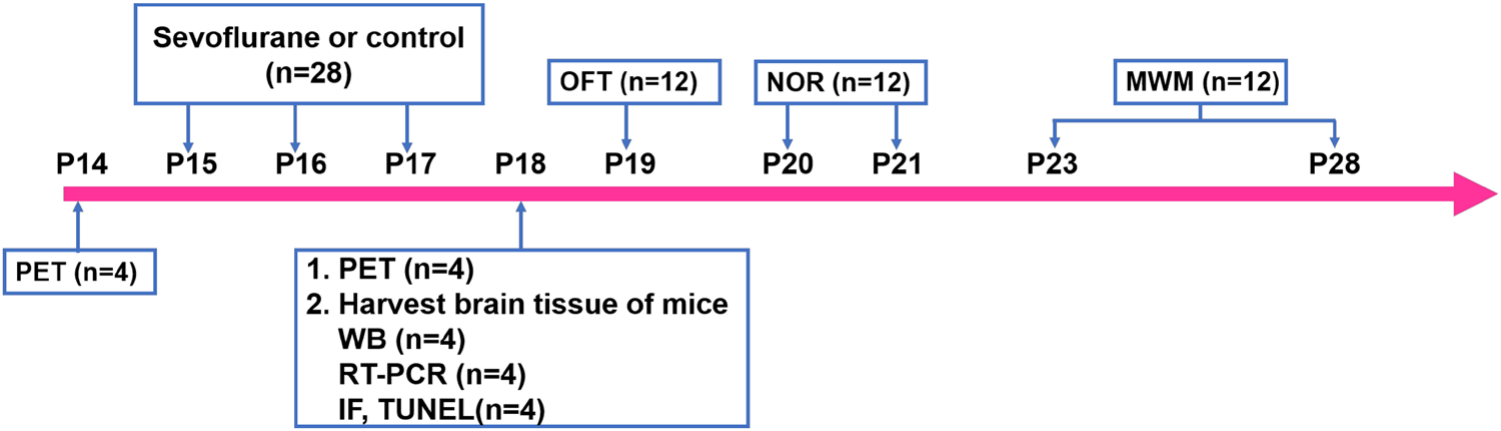
Illustration of the experimental timeline. Young mice (*n* = 28) were exposed to 3% sevoflurane or control for 2 h over 3 days at postnatal day (P) 15, P16, and P17. Each group (*n* = 4) of young mice was subjected to PET for measurement of cerebral glucose metabolism 24 h before the first exposure to sevoflurane on P14 and 24 h after the third exposure on P18. We harvested brain tissue (hippocampus and temporal lobe) 24 h after the last exposure to sevoflurane on P18 for WB (*n* = 4), RT-PCR (*n* = 4), and IF and TUNEL (*n* = 4) studies. Both groups of mice (*n* = 12) were used for an assessment of emotional reactivity using the OFT on P19 and an assessment of learning and memory function using the NOR from P20 to P21 and the MWM P23 to P28

Young (P14), adult (6-month-old) and old (22-month-old) healthy male C57BL/6 mice were purchased from Vital River Laboratory Animal Technology (China) (Dutta and Sengupta 2016). Different-aged mice were randomly assigned into two groups: a control (Con) group and a sevoflurane (Sev) group. Mice in the Sev group were exposed to 3% sevoflurane (Maruishi, Japan) in an air-O_2_ mixture 2 h daily for three days. The total gas flow was 2 l/min. We continually monitored the sevoflurane concentration, partial pressure of end-tidal carbon dioxide (ETCO_2_) using gas monitors (Draeger, Germany) and saturated oxygen in arterial blood (SpO_2_) using a pulse oximeter for animals (Kent, U.S.). Previous studies have shown that the administration of 3% sevoflurane for 2 h does not alter blood gas or brain blood flow in neonatal mice (Satomoto et al. 2009; Lu et al. 2010; Tao et al. 2014). The temperature of the anaesthetizing chamber was maintained at 37 °C by a heat plate. The young, adult and old mice were subjected to positron emission tomography (PET) for measurement of cerebral glucose metabolism 24 h before the first exposure to sevoflurane and 24 h after the third exposure to sevoflurane, and young mice were used for the subsequent experiments (Fig. 1). Each group of young mice was decapitated 24 h after the last sevoflurane exposure on P18, and brain tissue was harvested for use in Western blot (WB), real-time quantitative polymerase chain reaction (RT-PCR), immunofluorescence (IF), and terminal deoxynucleotidyl transferase dUTP nick end labelling (TUNEL) studies. Subsequently, the young mice were used for the open field test (OFT) on P19, the novel object recognition test (NOR) from P20 to P21 and the Morris water maze (MWM) from P23 to P28.

### Cell Cultures and Treatments

Hippocampal neuron cultures were prepared as previously described (Beaudoin et al. 2012). Briefly, the hippocampi of P0-P1 C57BL/6 mice were completely dissected on ice and digested with 1 mg/ml papain (Solarbio, China) for 20 min. After digestion was terminated with plating medium (87% DMEM + 10% FBS + 1% glutamine + 1% penicillin/streptomycin + 1% sodium pyruvate, all from Gibco, Thermo Fisher), neurons were plated on 6-well plates that were placed into an incubator containing 5% CO_2_ at a constant temperature of 37 °C. After 4 h of incubation, the plating medium was replaced with maintenance medium (96% neurobasal medium + 2% B-27 + 1% glutamine + 1% penicillin/streptomycin, all from Gibco), and half of the medium was changed after three days.

The PC12 cell line was obtained from the Shanghai Cell Bank of Type Culture Collection of Chinese Academy of Science. Cells were cultured in RPMI 1640 medium (HyClone, U.S.) with 10% horse serum (Solarbio), 5% FBS (Gibco), and 1% streptomycin/ penicillin (Gibco). After the PC12 cells were incubated for 24 h, 50 ng/ml nerve growth factor (NGF) (Solarbio) was used to induce differentiation. The following experiments were performed 4 days after NGF addition.

According to procedures for the anaesthetization of cell cultures (Matsuoka et al. 2001), the 6-well plates and a cell culture tube filled with double-distilled water for humidity were placed into an anaesthesia chamber. The chamber was put into a water bath at 37 °C. Cells were exposed to 3% sevoflurane 2 h daily for three consecutive days. The following experiments were performed 24 h after the third exposure to sevoflurane.

### GLUT3 Overexpression in Neural Cultures

The lentiviral vectors LV5-EF-GFP-puro (LV5) carrying a green fluorescent protein (GFP) and puromycin-resistance gene (puro) expression cassette were designed as negative controls, and the GLUT3 gene was cloned into LV5 vectors (LV5-GLUT3), which were supplied by GenePharma Co., Ltd., China. Our preliminary results showed that a multiplicity of infection (MOI) of 100 and a puromycin (GenePharma) concentration of 5 ng/ml were the best parameters for further experiments. After 5 days of incubation in 6-cm plates and after the PC12 cells were 60% confluent, 200 µl containing 10^8^ TU LV5-GLUT3/ml was added to each well. GFP expression was observed under a microscope 36 h after transfection to identify the transfection efficiency. Puromycin (5 ng/ml) was used to select PC12 cells stably overexpressing GLUT3 for subsequent experiments, and primary neurons were not used for this step.

### [18F] Fluorodeoxyglucose (18F-FDG) Positron Emission Tomography (PET) Imaging

Three different-aged mice were intraperitoneally injected with approximately 250 ± 10 μCi 18F-FDG after 12 h of fasting. After a 45-min uptake period, images were obtained with a static scanning pattern (10 min) by a Trans-PET BioCaliburn 700 system (Raycan Technology Co., Ltd., Suzhou, China). The animals were subjected to PET scanning again 24 h after three days of sevoflurane exposure. Forty-five minutes are required for the uptake and metabolic trapping of 18-FDG in organisms, and the PET scan data indicated regional 18-FDG uptake and metabolism during the 45-min uptake period (Alkire et al. 1997). According to the principals of 18F-FDG metabolism described above, the final images show glucose metabolism 24 h after anaesthesia. The images were reconstructed using the three-dimensional (3D) ordered subset expectation–maximization (OSEM) method with a voxel size of 0.5 × 0.5 × 0.5 mm^3^. Volume-of-interest (VOI) analysis was conducted using the AMIDE software package (Free Software Foundation, Inc., Boston, Massachusetts, USA). The mean standardized uptake value (SUV) in the brain regions was measured using the following formula: mean pixel value with the decay-corrected region-of-interest activity (μCi/kg)/(injected dose [μCi]/weight [kg]).

### Cell Viability Assay

A CCK-8 kit (BS350A, Biosharp, China) was used to assess primary neural viability in 96-well plates. Ten microliters of CCK-8 solution was added to each well. After incubation for 2 h, cell viability was analysed by an EnSpire multimode plate reader (Perkin-Elmer, U.S.) at a wavelength of 450 nm. The results are presented as a percent of the control results.

### Western Blotting

Tissue and cellular proteins were extracted using RIPA total protein lysate (Aspen, China) following the manufacturer’s instructions. Proteins from each sample were separated by electrophoresis on 8–15% SDS-PAGE gels and transferred onto PVDF membranes (Aspen). After incubation with WB-specific blocking solution (5% skimmed milk powder (AS1033, Aspen, China) diluted in TBST), the PVDF membranes were separately incubated with anti-GLUT1 (1:1000, ab652, Abcam), anti-GLUT4 (1:2000, ab654, Abcam), anti-(extracellular) GLUT3 (1:1000, AGT-023, Alomone), anti-Bax (1:800, sc-7480, Santa Cruz), anti-Bcl-2 (1:800, sc-7382, Santa Cruz), anti-caspase-3 (cleaved) (1:100, AB3623, Millipore), anti-poly (ADP-ribose) polymerase (PARP) (cleaved) (1:1000, 5625, Cell Signaling Technology), and anti-β-actin (1:10,000, Aspen) antibodies at 4 °C overnight. After the blots were washed, they were incubated with HRP-conjugated secondary antibody for 1.5 h and detected by a chemiluminescent imaging system (Tanon, China).

### Real-Time Quantitative Polymerase Chain Reaction

Total RNA was isolated with the TRIpure Total RNA Extraction Reagent (ELK Biotechnology, China), and the concentration and purity of the total RNA were measured by a NanoDrop 2000 (Thermo Fisher, U.S.). Reverse transcription was performed using a 1st Strand Synthesis Kit (ELK Biotechnology). The following primers were designed in advance: mouse-GLUT3, (F) 5′-ACCTCCAACTTTCTGGTCGG-3′ and (R) 5′-CTTTGGTCTCCGGGACTTTG-3′. RT-PCR was performed using SYBR Green PCR SuperMix (ELK Biotechnology) in a real-time PCR system (CFX96 Touch, Bio-Rad, U.S.). Levels of the GLUT3 gene were quantified using the ΔΔCt method with β-Actin as an internal control.

### Immunofluorescence

Paraffinized slides containing brain tissues were washed and incubated with IF-specific blocking solution (10% goat serum (AR0009, Boster, China) diluted in PBS) for 30 min. The slides were incubated with anti-GLUT3 (1:150, AGT-023, Alomone) overnight at 4 °C before being washed three times with PBS and then incubated with the Cy3 goat anti-rabbit antibody (Aspen). The slides were imaged using an Aperio VERSA 8 microscope (Leica, Germany), and the images were analysed using imaging software.

Neurons were fixed on coverslips with 4% paraformaldehyde for 30 min. After incubation with 0.5% Triton X-100 (Aspen) for 20 min, the coverslips were incubated in IF-specific blocking solution for 1 h at room temperature. Cells were incubated with anti-GLUT3 (1:150, AGT-023, Alomone) overnight at 4 °C. After the cells were washed with PBS, they were incubated with the Cy3 goat anti-rabbit antibody (Aspen) for 1 h at room temperature. The signal from the secondary antibody was captured using an IX73 fluorescence microscope (Olympus, Japan), and the images were analysed using imaging software.

### Terminal Deoxynucleotidyl Transferase dUTP Nick End Labelling

Apoptosis in slides containing brain tissues was detected using a Roche In Situ Cell Death Detection kit (11684795910; Sigma Aldrich) according to the manufacturer’s instructions, followed by propidium iodide nuclear counterstaining. The number of TUNEL-positive cells in the hippocampus and temporal lobe were counted at × 200 magnification with an Aperio VERSA 8 microscope (Leica, Germany).

### Flow Cytometry

To detect the surface expression of GLUT3, we harvested PC12 cells from 6-well plates using 0.05% trypsin 24 h after the third exposure to sevoflurane. The PC12 cells were washed twice with pre-chilled blocking solution and incubated with anti-(extracellular) GLUT3 (1:100, AGT-023, Alomone) at 4 °C for 1 h. After the cells were washed, they were incubated with phycoerythrin (PE)-Cy5 goat anti-rabbit IgG (Bioss, China) for 30 min. The samples were immediately subjected to flow cytometry on a CytoFLEX (Beckham Coulter, U.S.).

To detect the apoptosis rate of the PC12 cells, we analysed early and late apoptosis in cells by an Annexin V-PE Apoptosis Analysis Kit (AO2001-09A-G, Sungene Biotech) according to the manufacturer’s protocol. Briefly, PC12 cells were harvested, washed with cold PBS and then suspended in 1 × binding buffer. After the cells were resuspended, 100 µl of cells (1 × 105) was added to each labelled tube and incubated with 5 µl Annexin V-PE for 10 min. Then, 5 µl 7-AAD was added to each tube for 5 min. After PBS was added to a volume of 500 µl, the PC12 cells were subjected to flow cytometry.

### Open Field Test

Mice were transported to the testing room at least 1 h prior to the experiment to acclimate to the experimental room. The lights were kept bright, the temperature was kept warm, and the ambient environment remained quiet. Each mouse was randomly placed in the centre of the box (40 cm × 40 cm × 40 cm) and allowed to explore the apparatus for 5 min. A tracking camera recorded the activity of the mice in the box. When the mouse was removed from the box, the box was wiped clean with alcohol to avoid leaving olfactory cues.

### Novel Object Recognition Test

The NOR test was carried out according to the experimental scheme of Bevins et al. (Bevins and Besheer 2006). Before the experiment, each mouse was randomly placed in a box (40 cm × 40 cm × 40 cm) for 3 min to adapt to the new environment. After acclimation, the smell and waste were removed with 70% alcohol. The mice were randomly placed into the box to explore two similar objects for 5 min each. Twenty-four hours later, one object was replaced with a novel object with a different shape but the same other properties. Each mouse was allowed to explore for 5 min. A computer system recorded the movement of each mouse.

### Morris Water Maze

To avoid exhaustion of the young P23 mice and difficulty in completing the task, we employed a small white pool (diameter of 0.8 m, height of 0.5 m) according to previous procedures (Rudy et al. 1987; Jett et al. 1997). The pool was divided into four quadrants: the east (E), west (W), south (S), and north (N) quadrants, and an initial quadrant was randomly selected. The depth of the water was 0.4 m, and the water temperature was maintained at 23 °C to keep the mice warm. A glass platform (10 cm in diameter) was placed 2 cm under the surface of the water and thus completely hidden from view. The top of the pool was equipped with a small camera to record the movement of the mice. Each mouse was randomly and gently placed into each quadrant of the pool every day during five consecutive days of training. Each mouse was allowed to search for the platform for 60 s, and then the mice were given a 30-sec break on the platform (Rudy et al. 1987; Jett et al. 1997). If the mice could not find the platform, researchers guided the mice to the platform to rest for 30 s. On the sixth day, during which the escape platform was removed, the probe trial lasted 60 s.

### Statistical Analysis

Data were analysed by SPSS statistics version 21.0 (SPSS Inc., Armonk, USA) software and are presented as the mean ± s.e.m. A paired Student’s *t* test was used to determine the difference in the PET data from the young, adult and old mice before and after sevoflurane exposure. Two-way analysis of variance (ANOVA) followed by the Bonferroni test was used to analyse the differences in escape latency measured in the MWM between the mice exposed to sevoflurane and the mice in the control group (Tao et al. 2014). ANOVA with post hoc Tukey's test was used to determine the significance of differences in the other data among groups. *P* < 0.05 indicated statistical significance.

## Results

### Sevoflurane Reduces Brain Glucose Metabolism in Young Mice

**Fig. 2:**
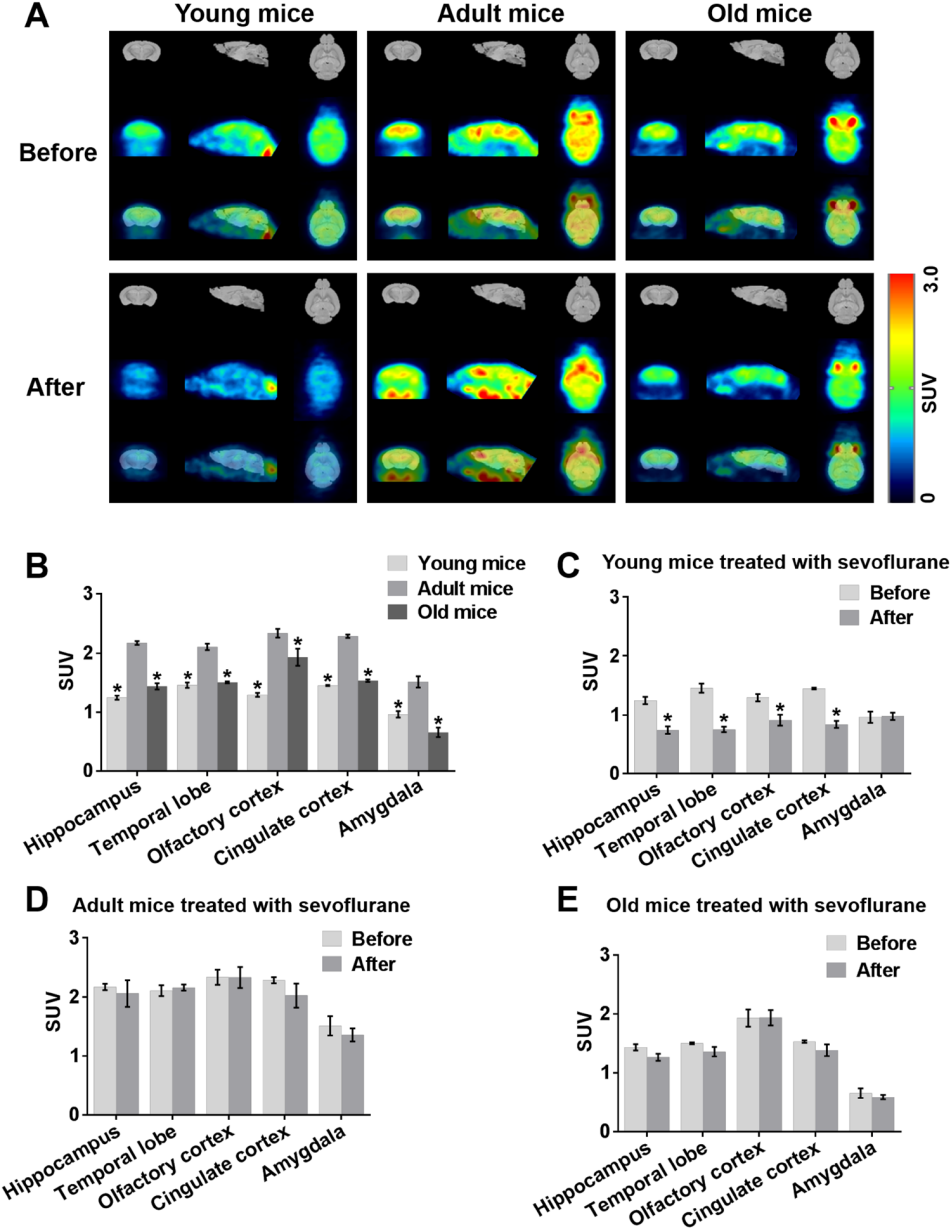
Sevoflurane reduces brain glucose metabolism in young mice. **a** PET images illustrating local cerebral glucose metabolism in young, adult and old mice 24 h before the first exposure to sevoflurane (upper panel) and 24 h after the third exposure (lower panel). *n* = 4 for each group. **b** Representative quantification of glucose metabolism in five brain regions from three different-aged mice 24 h before the first exposure to sevoflurane. *P < 0.05 versus adult mice. **c, d, e** Representative quantification of glucose metabolism in five brain regions from young, adult and old mice 24 h before the first exposure to sevoflurane and 24 h after the third exposure. *P < 0.05 versus cerebral glucose metabolism in young mice 24 h before the first exposure to sevoflurane. The data are presented as the mean ± s.e.m

To disclose age-related changes in regional glucose metabolic activity among young, adult and old mice using predefined SUV, we monitored specific brain regions by 18F-FDG PET. Glucose metabolism in the hippocampus, temporal lobe, olfactory cortex, cingulate cortex and amygdala was lower in young and old mice than in adult mice (Fig. 2a, b). To investigate the actual role of anaesthesia on cerebral glucose metabolism in different-aged mice, it is very important to know whether the cognitive dysfunction after sevoflurane is due to cerebral metabolism. Therefore, in this study, resting brain glucose uptake in young, adult and old mice was measured at normal baseline and 24 h after the third exposure to sevoflurane. Interregional metabolic correlation on 18F-FDG PET has been shown to reflect interregional covariance patterns of neuronal activities (Lee et al. 2008). Brain glucose metabolism 24 h after the third exposure to sevoflurane did not differ from that 24 h before the first exposure in adult mice or old mice (Fig. 2a, d, e). Surprisingly however, in young mice, glucose metabolism in the hippocampus, temporal lobe, olfactory cortex, and cingulate cortex but not amygdala 24 h after the third exposure to sevoflurane was lower than that 24 h before the first exposure (Fig. 2a, c).

### Sevoflurane Impairs Learning and Memory in Young Mice

**Fig. 3:**
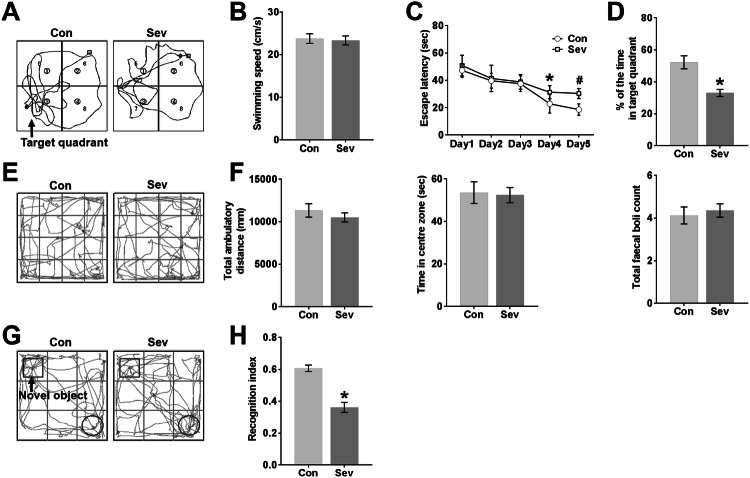
Sevoflurane impairs learning and memory in young mice. **a** Swimming tracks during the probe trial on P28. The arrow denotes the quadrant in which the escape platform was previously placed during MWM training from P23 to P27. **b** The average swimming speed during the probe trial on P28. **c** Escape latency (the time to locate the escape platform) during MWM training from P23 to P27. On P26, *P < 0.05 versus the Con group. On P27, ^#^*P* < 0.05 versus the Con group. **d** Percent time spent in the target quadrant during the probe trial on P28. *P < 0.05 versus the Con group. **e** Tracks in the OFT. **f** Total ambulatory distance, time spent in the centre zone, and total faecal boli during the OFT. These are effective indicators of emotional activity in mice. **g** Tracks in the testing phase of the NOR test. The arrow denotes the novel object moved to a novel location in the testing phase. **h** RI in the testing phase of the NOR test. RI = the time devoted to the novel object/the total time devoted to the familiar and novel objects. *P < 0.05 versus the Con group. *n* = 12 in the Con group, *n* = 11 in the Sev group. The data are presented as the mean ± s.e.m

The MWM and NOR tests are generally used to detect spatial learning, memory and familiarity memory related to the hippocampus and extensive cortices (Morris, 1984; Bevins and Besheer, 2006; Vorhees and Williams, 2006; Eichenbaum et al. 2007; Squire et al. 2007), and the OFT is typically used to measure emotional reactivity of the amygdala (Archer 1981; Davis 1992). There was no difference in average swimming speed between the Con group and the Sev group in the MWM test (Fig. 3b). Sevoflurane anaesthesia increased the escape latency time for mice to locate the platform in the MWM pool compared with the control conditions on P26 and P27 (Fig. 3c). Sevoflurane anaesthesia also reduced the time in the target quadrant during the probe trial on P28 compared with control conditions (Fig. 3d). The swimming tracks of the Sev group showed less of a bias towards the target quadrant that previously contained the escape platform during the probe trial than did those of the Con group (Fig. 3a), indicating spatial memory loss caused by sevoflurane exposure.

To study whether the anxiety-related behaviour of mice exposed to sevoflurane was affected, mice underwent the OFT. Both groups of mice moved more around the periphery, avoiding the central zone (Fig. 3e). In addition, anaesthetized mice and control mice did not differ significantly in total ambulatory distance, time spent in the centre zone, or faecal boli deposits in the OFT (Fig. 3f), showing that the emotional activity in young mice was not affected by sevoflurane anaesthesia. To examine responses to a novel environment, young mice exposed to sevoflurane were assayed in the NOR test. The results of the NOR paradigm are influenced by both hippocampal and cortical lesions (Antunes and Biala 2012). The movement of the mice in the testing phase was continually tracked, and the tracks showed that anaesthetized mice spent less time exploring the novel object in the testing phase than did control mice (Fig. 3g). The percentage of time spent exploring the novel object relative to the total time spent exploring both objects, presented as the recognition index (RI), is used as a measure of NOR. Compared to the Con group, the Sev group exhibited a significantly lower RI in young mice (Fig. 3h), reflecting a learning and memory impairment induced by multiple sevoflurane exposures.

### Sevoflurane Decreases GLUT3 Protein Expression in the Hippocampus and Temporal Lobe

**Fig. 4:**
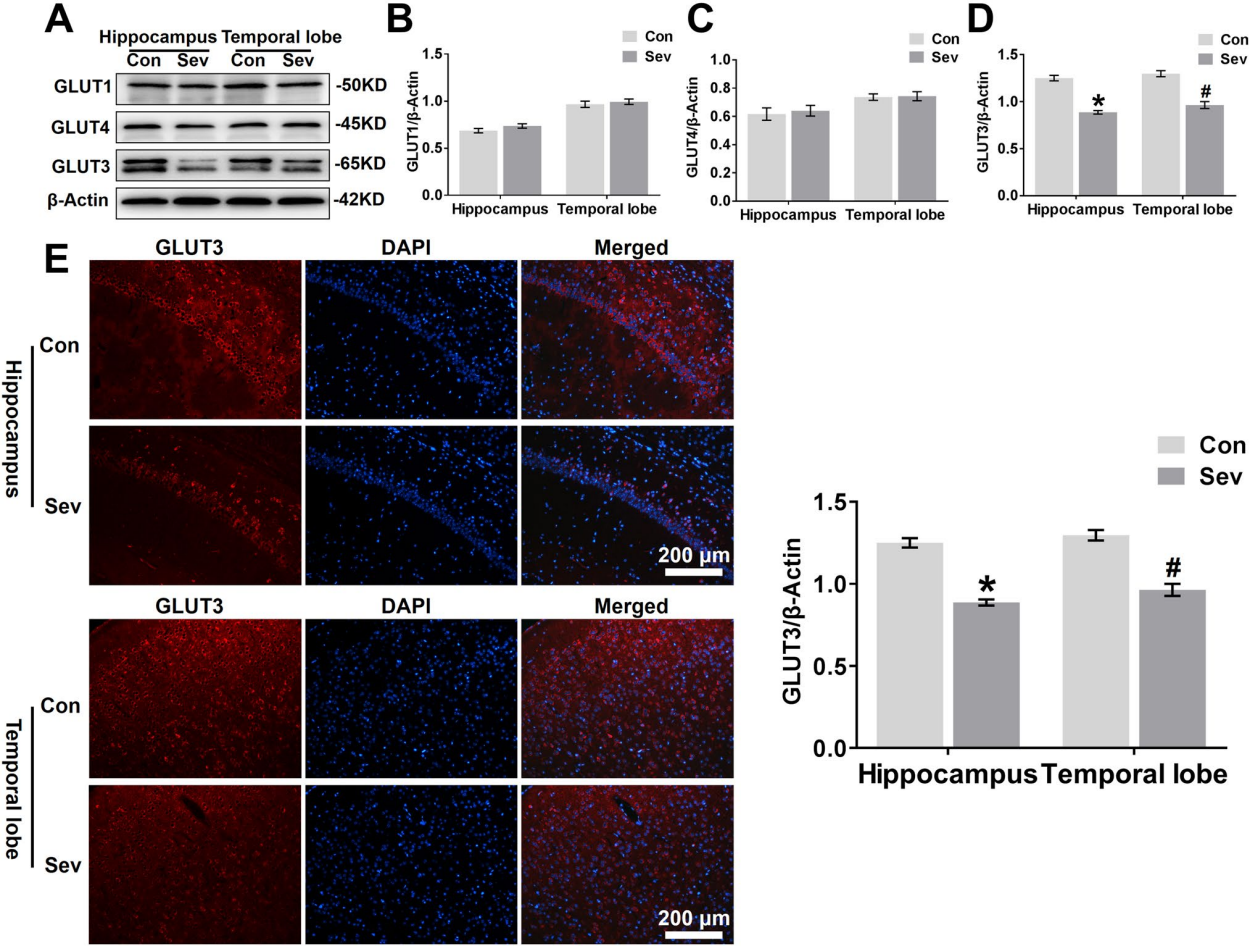
Sevoflurane decreases GLUT3 protein expression in the hippocampus and temporal lobe. **a** WB analysis of GLUT1, GLUT4, and GLUT3 protein expression in the hippocampus and temporal lobe of young mice 24 h after the last exposure to sevoflurane. *n* = 4 for each group. **b, c, d** Histograms showing the quantification of GLUT1, GLUT4, and GLUT3 blots in the hippocampus and temporal lobe. In the hippocampus, *P < 0.05 versus the Con group. In the temporal lobe, ^#^*P* < 0.05 versus the Con group. **e** Fluorescent images showing GLUT3 expression in neurons of the hippocampal CA1 area and temporal lobe (left panel). Quantification of GLUT3 intensity in the CA1 region of the hippocampus and temporal lobe 24 h after the last exposure to sevoflurane (right panel). In the CA1 region of the hippocampus, *P < 0.05 versus the Con group. In the temporal lobe, ^#^*P* < 0.05 versus the Con group. n = 4 for each group. The data are presented as the mean ± s.e.m

Glucose metabolism in neurons is closely related to glucose transport, and we sought to determine whether sevoflurane suppresses glucose transporters, contributing to the decrease in glucose utilization in the hippocampus and temporal lobe in young mice. As shown in Fig. 4a and d, young mice exposed to sevoflurane had a lower level of GLUT3 protein expression but not GLUT1 (Fig. 4a and b) or GLUT4 protein expression (Fig. 4a and c) in the hippocampus and temporal lobe than control mice. Immunofluorescent staining also revealed an overall significant decrease in GLUT3 levels in the CA1 region of the hippocampus and temporal lobe of young animals exposed to anaesthesia (Fig. 4e).

**Fig. 5:**
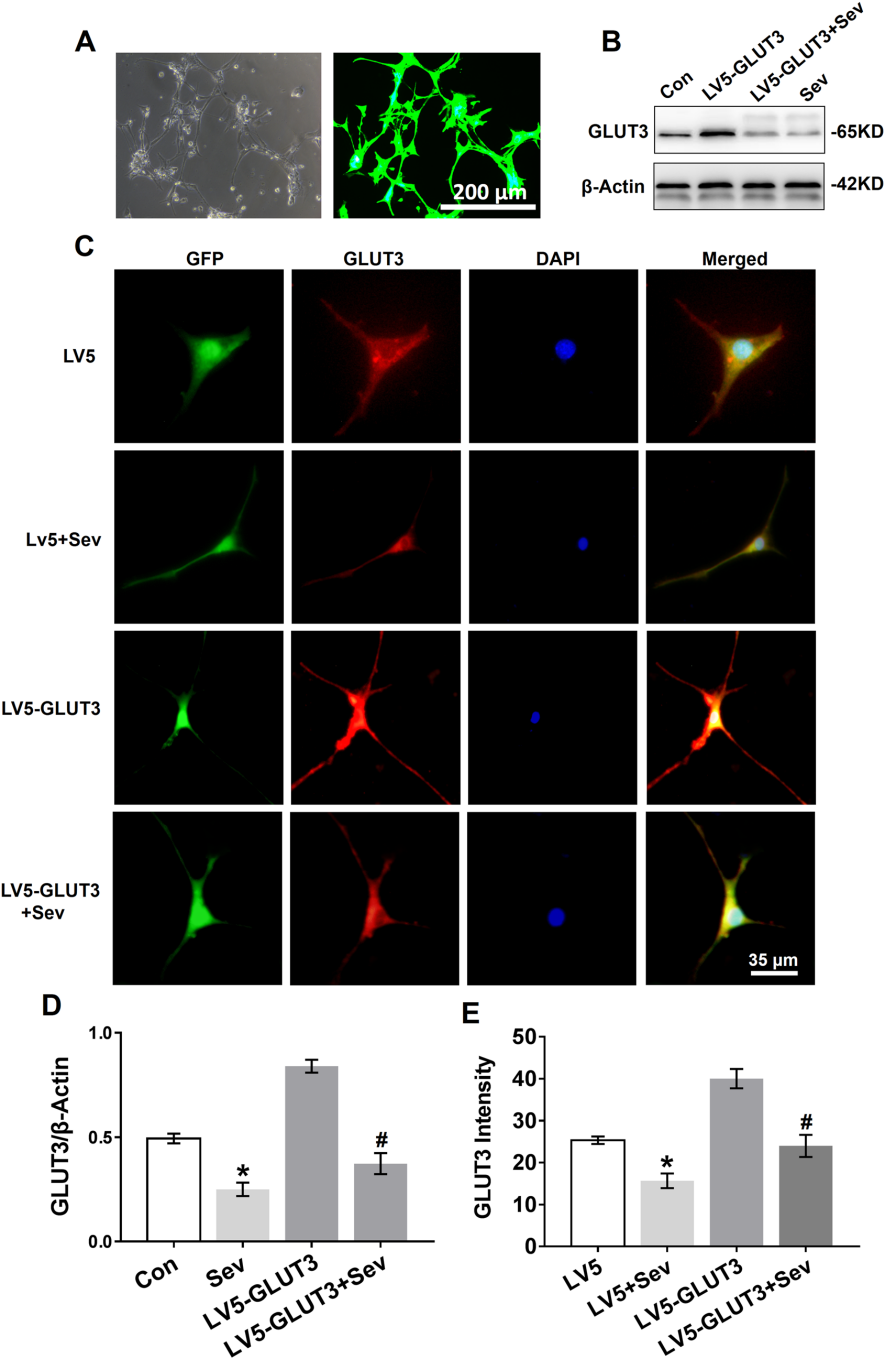
Sevoflurane reduces GLUT3 protein expression in primary hippocampal neurons. **a** Primary hippocampal neurons were transfected with LV5-GLUT3. The transfection efficiency was examined by GFP expression (green) 36 h post infection, which was analysed by phase contrast (left panel) and fluorescence microscopy (right panel). **b** WB analysis of GLUT3 protein expression in primary hippocampal neurons 24 h after the last exposure to sevoflurane. Neurons were normally cultured (the Con group), only transfected with LV5-GLUT3 (the LV5-GLUT3 group), transfected with LV5-GLUT3 and then exposed to sevoflurane (the LV5-GLUT3 + Sev group), and only exposed to sevoflurane (the Sev group). **c** Immunofluorescence of GLUT3 expression in the somata and dendrites of primary hippocampal neurons 24 h after the last exposure to sevoflurane. The four groups: the LV5 group (neurons transfected with LV5 empty vectors), the LV5 + Sev group (neurons transfected with LV5 empty vectors and then exposed to sevoflurane), the LV5-GLUT3 and the LV5-GLUT3 + Sev group. **d** Histograms showing the results of GLUT3 blots in neurons among the four groups. *n* = 5 for each group. *P < 0.05 versus the Con group. ^#^*P* < 0.05 versus the LV5-GLUT3 group. **e** Histograms showing the intensity of GLUT3 in the four treatment groups. *n* = 5 for each group. *P < 0.05 versus the LV5 group. ^#^*P* < 0.05 versus the LV5-GLUT3 group. The data are presented as the mean ± s.e.m

Next, we investigated whether sevoflurane inhibits GLUT3 protein expression in vitro by transfecting primary hippocampal neurons with lentivirus overexpressing GLUT3. The lentiviral transfection efficiency was assessed by exploitation of GFP expression, which showed that the lentiviral system carrying the GLUT3 gene had a high transfection yield (Fig. 5a). We found that sevoflurane decreased GLUT3 protein expression in neurons 24 h after the last exposure to sevoflurane (Fig. 5b, d). As expected, sevoflurane significantly decreased GLUT3 protein expression in neurons overexpressing GLUT3 (Fig. 5b, d). Moreover, immunofluorescent staining revealed GLUT3 protein expression in the somata and dendrites of primary hippocampal neurons. As shown in Fig. 5c, e, sevoflurane decreased GLUT3 protein expression in neurons infected with LV5 empty vectors and LV5-GLUT3.

### Sevoflurane Inhibits GLUT3 Surface Expression and GLUT3 mRNA Expression in Neurons

**Fig. 6:**
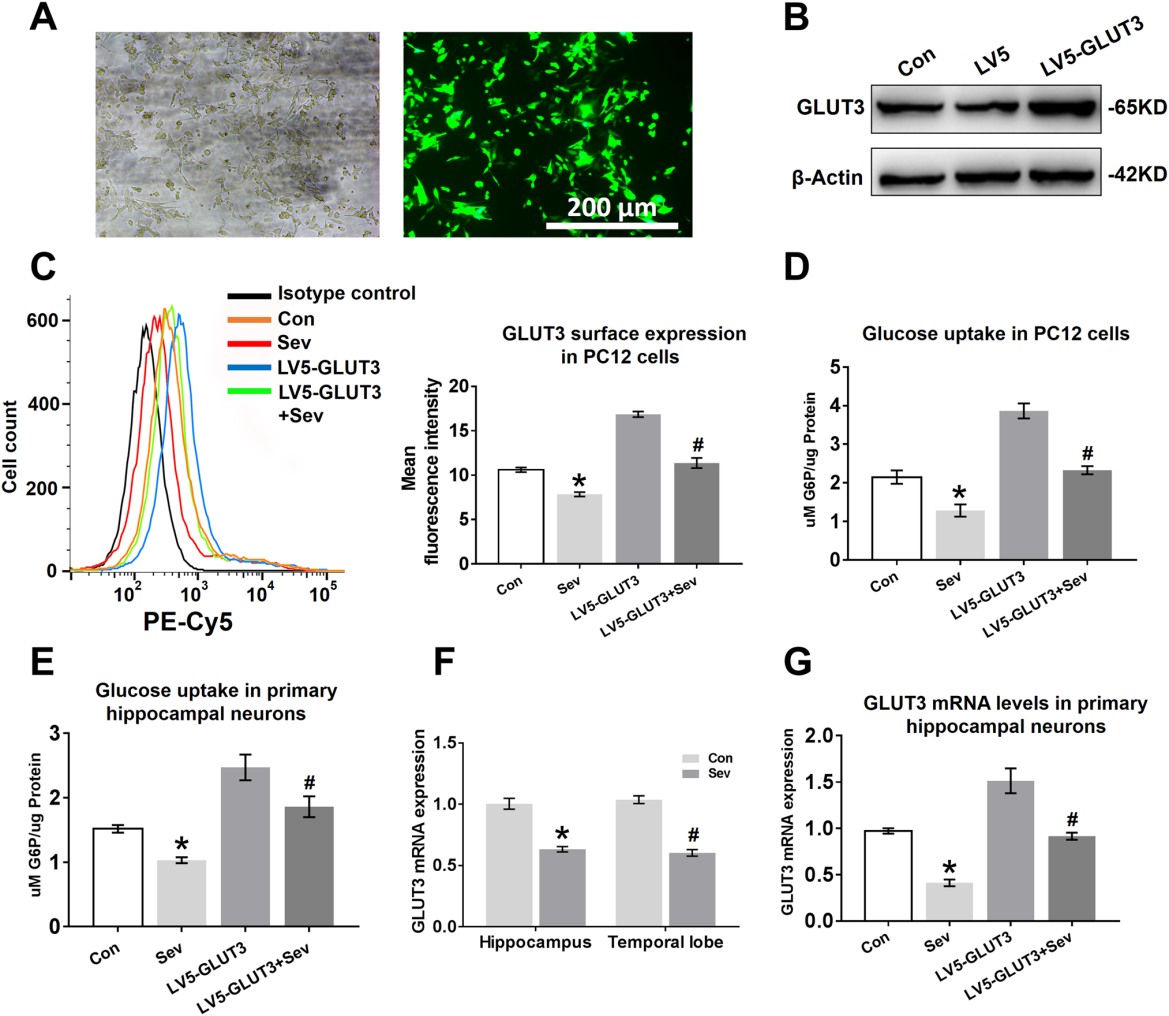
Sevoflurane inhibits GLUT3 surface expression and GLUT3 mRNA expression in neurons. **a** PC12 cells were transfected with LV5-GLUT3. The transfection efficiency was examined by GFP expression (green) 36 h post infection, which was analysed by phase contrast (left panel) and fluorescence microscopy (right panel). **b** The expression levels of GLUT3 protein in PC12 cells infected with LV5-GLUT3 were determined by WB analysis. PC12 cells were normally cultured (the Con group), transfected with LV5 empty vectors (the LV5 group), or transfected with LV5-GLUT3 (the LV5-GLUT3 group). **c** Flow cytometry analysis of GLUT3 surface expression in PC12 cells 24 h after the last exposure to sevoflurane. Four groups: the Con group, the Sev group (PC12 cells only exposed to sevoflurane), the LV5-GLUT3 group and the LV5-GLUT3 + Sev group (PC12 cells transfected with LV5-GLUT3 and then exposed to sevoflurane). *n* = 5 for each group. *P < 0.05 versus the Con group. ^#^*P* < 0.05 versus the LV5-GLUT3 group. **d** Representative G6P concentrations showing glucose uptake in PC12 cells 24 h after the last exposure to sevoflurane. *n* = 5 for each group. *P < 0.05 versus the Con group. ^#^*P* < 0.05 versus the LV5-GLUT3 group. **e** Representative G6P concentration showing glucose uptake in primary hippocampal neurons 24 h after the last exposure to sevoflurane. *n* = 5 for each group. *P < 0.05 versus the Con group. ^#^*P* < 0.05 versus the LV5-GLUT3 group. **f** Representative quantification of GLUT3 mRNA expression in the hippocampus and temporal lobe 24 h after the last exposure to sevoflurane. *n* = 4 for each group. In the hippocampus, *P < 0.05 versus the Con group. In the temporal lobe, ^#^*P* < 0.05 versus the Con group. **g** Representative quantification of GLUT3 mRNA expression in primary hippocampal neurons 24 h after the last exposure to sevoflurane. *n* = 5 for each group. *P < 0.05 versus the Con group. ^#^*P* < 0.05 versus the LV5-GLUT3 group. The data are presented as the mean ± s.e.m

To investigate whether sevoflurane has an impact on GLUT3 surface expression, we replaced primary hippocampal neurons with PC12 cells for flow cytometry experiments. We first measured the transfection efficiency and exogenous GLUT3 protein expression in PC12 cells transfected with LV5-GLUT3. The GFP expression observed by fluorescence microscopy showed a high level of transfection efficiency 36 h post infection (Fig. 6a). Furthermore, the expression of GLUT3 protein was almost twofold higher in PC12 cells transfected with LV5-GLUT3 than in cells transfected with LV5 empty vectors (Fig. 6b). Next, we determined the effect of sevoflurane anaesthesia on GLUT3 translocation to the cell surface under conditions of non-transfection or transfection. As shown in Fig. 6c, sevoflurane decreased GLUT3 surface expression in PC12 cells; although GLUT3 surface expression was enhanced by transfection, GLUT3 surface expression decreased after sevoflurane exposure (Fig. 6c). We further assessed whether the decline in GLUT3 surface expression induced a decrease in glucose uptake in PC12 cells. G6P analysis showed that sevoflurane anaesthesia reduced the levels of G6P in PC12 cells (Fig. 6d). Furthermore, the elevated glucose utilization in the LV5-GLUT3 group was diminished by multiple sevoflurane exposures (Fig. 6d). Although we could not detect the surface expression of GLUT3 in primary hippocampal neurons, sevoflurane diminished glucose uptake in hippocampal neurons, and overexpression of GLUT3 in hippocampal neurons ameliorated the reduction in glucose uptake caused by sevoflurane anaesthesia (Fig. 6e), suggesting that sevoflurane anaesthesia reduces the surface expression of GLUT3 in primary hippocampal neurons.

GLUT3 protein expression was significantly reduced in vivo (Fig. 4a, e) and in vitro (Fig. 5b, c) 24 h after the last exposure to sevoflurane. To determine whether sevoflurane acted on GLUT3 gene expression, we analysed GLUT3 mRNA expression in vitro and in vivo 24 h after the last exposure to sevoflurane using RT-PCR. Young mice exposed to sevoflurane had lower levels of GLUT3 mRNA in the hippocampus and temporal lobe than young mice in the Con group (Fig. 6f). Similarly, sevoflurane exposure also reduced GLUT3 mRNA levels in primary hippocampal neurons (Fig. 6g), and even neurons infected with LV5-GLUT3 exhibited a decrease in GLUT3 mRNA expression 24 h after the last exposure to sevoflurane.

### Sevoflurane Induces Neural Apoptosis in the Hippocampus and Temporal Lobe

**Fig. 7:**
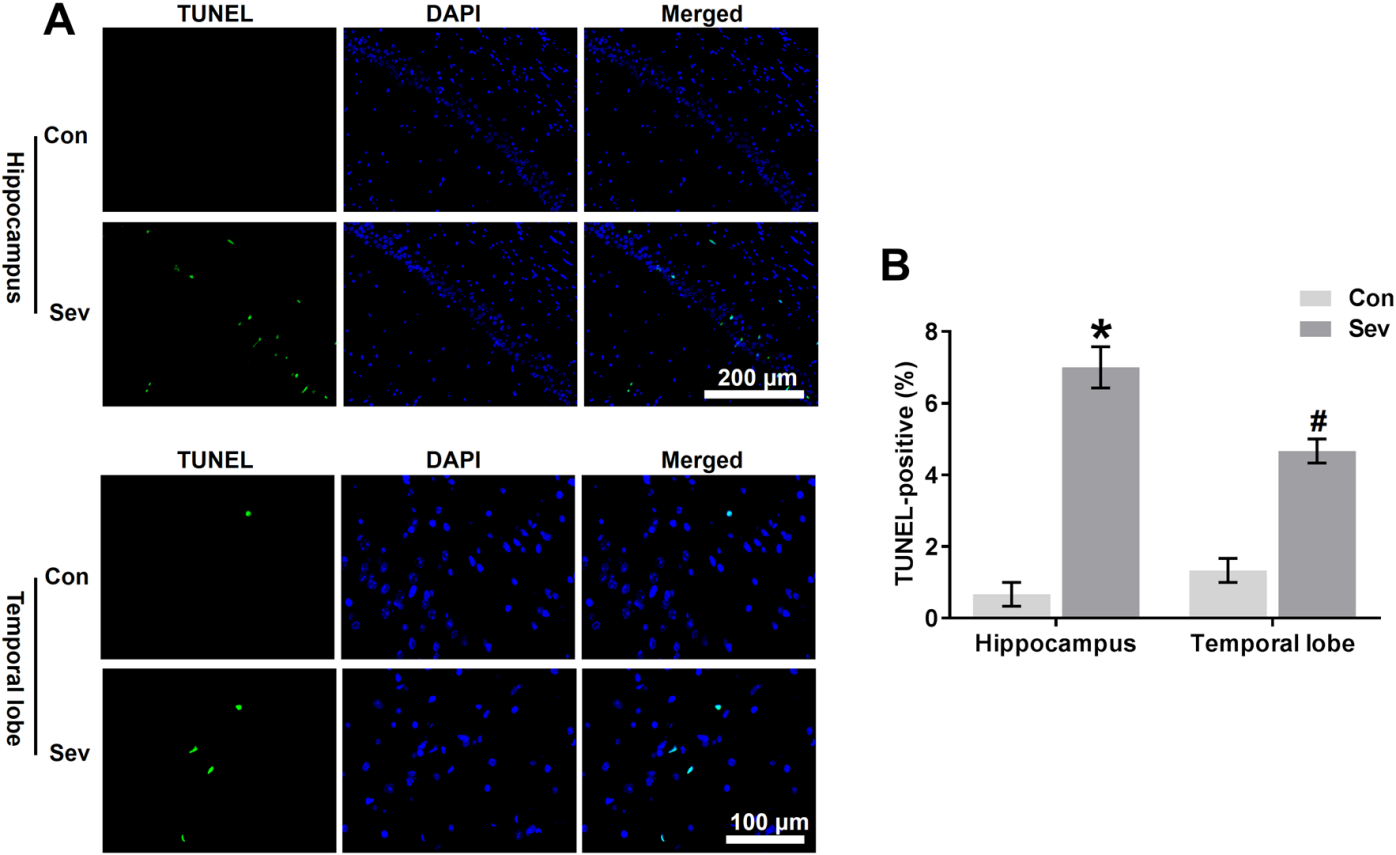
Sevoflurane induces neural apoptosis in the hippocampus and temporal lobe. **a** Representative images showing apoptosis in the CA1 region of the hippocampus and temporal lobe of young mice 24 h after the third exposure to sevoflurane. Apoptosis was indicated by TUNEL immunofluorescence (green), and nuclei were stained with DAPI (blue). **b** Representative quantification of TUNEL-positive cells in the CA1 region of the hippocampus and temporal lobe. *n* = 4 for each group. In the CA1 region of the hippocampus, *P < 0.05 versus the Con group. In the temporal lobe, ^#^*P* < 0.05 versus the Con group. The data are presented as the mean ± s.e.m

**Fig. 8:**
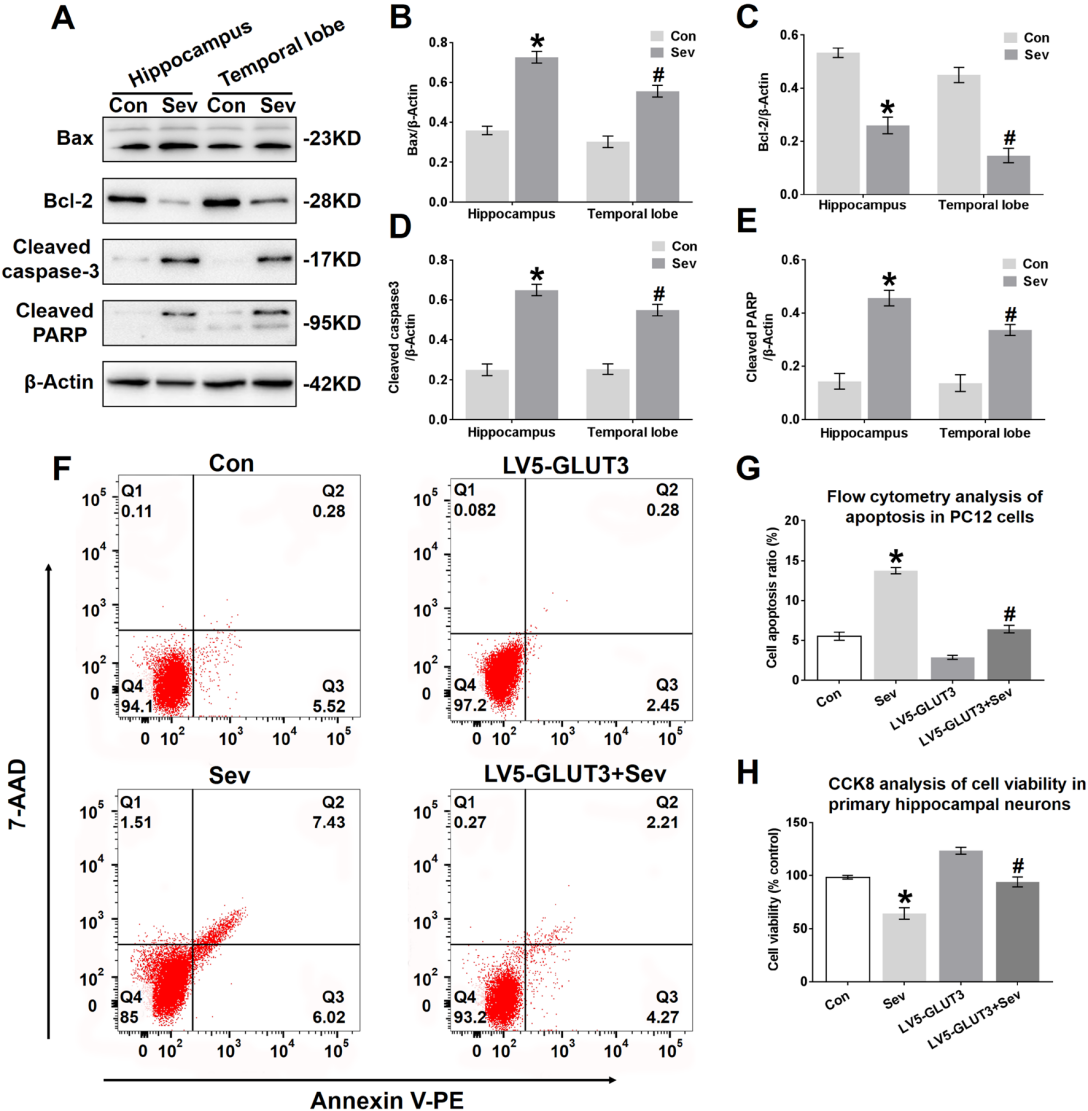
Sevoflurane induces neural apoptosis in the hippocampus and temporal lobe. **a** Representative immunoblots showing the protein expression of Bax, Bcl-2, cleaved caspase-3 and cleaved PARP in the hippocampus and temporal lobe of young mice 24 h after the last exposure to sevoflurane. *n* = 4 for each group. **b, c, d, e** Histograms showing the results of Bax, Bcl-2, cleaved caspase-3, and cleaved PARP blots in the hippocampus and temporal lobe. *P < 0.05 versus the Con group. In the temporal lobe, ^#^*P* < 0.05 versus the Con group. **f** Representative images showing the flow cytometry analysis of the apoptosis ratio in PC12 cells 24 h after the last exposure to sevoflurane. Early apoptotic populations (PE^+^7-AAD^−^cells) are in the lower-right quadrant; late apoptotic cells (PE^+^7-AAD^+^) are in the upper-right quadrant in each dot plot. **g** Representative quantification of the flow cytometry analysis of the apoptosis ratio of PC12 cells 24 h after the last exposure to sevoflurane. **h** Representative CCK-8 assay showing cell viability in primary hippocampal neurons 24 h after the last exposure to sevoflurane. *n* = 5 for each group. *P < 0.05 versus the Con group. ^#^*P* < 0.05 versus the LV5-GLUT3 group. The data are presented as the mean ± s.e.m

A decline in glucose transport causes apoptosis in a number of different cell types (Richter et al. 1996, Mueckler 2000). The data described above indicated that sevoflurane induces a decrease in glucose transport into neurons (Fig. 6d, e). Furthermore, we evaluated the effects of sevoflurane on apoptosis in the hippocampus and temporal lobe of young mice. Young mice exposed to sevoflurane had more TUNEL-positive cells in the hippocampal CA1 region and temporal lobe than mice in the Con group (Fig. 7a, b). One of the key regulatory steps of apoptosis is controlled by the expression of antiapoptotic and proapoptotic proteins of the Bcl-2 family, which determine the susceptibility to apoptosis (Gross et al. 1999). Our results showed that sevoflurane increased Bax levels in the hippocampus and temporal lobe of young mice (Fig. 8a, b), whereas Bcl-2 levels were decreased (Fig. 8a, c), indicating that sevoflurane predisposed the immature brain to neuroapoptosis. Next, we examined the involvement of caspases in cleaving a variety of substrates as caspase-3 activation is considered an indicator of the apoptotic process (Porter and Janicke 1999). Our immunoblots showed increased expression of activated caspase-3 in the hippocampus and temporal lobe 24 h after the last exposure to sevoflurane (Fig. 8a, d). Once caspase-3 is activated, many cellular proteins are cleaved, including PARP. Our results also demonstrated that sevoflurane exposure elevated the levels of cleaved PARP (Fig. 8a, e), a downstream substrate of the caspase cascade and a reliable marker of apoptosis (Boulares et al. 1999).

To further investigate whether sevoflurane affects neural apoptosis, the neural apoptosis rate and cell viability in PC12 cells and primary hippocampal neurons, respectively, were determined. Phosphatidylserine (PS) externalization is one of the early biomarkers of apoptosis. Fluorochrome-labelled (PE) Annexin V is a probe with high affinity for PS designed for the early detection of apoptosis. Due to the loss of cell integrity during late apoptosis, 7-amino-actinomycin D (7-AAD) can enter late apoptotic cells to stain DNA. Therefore, staining with PE in conjunction with 7-AAD dye helps to identify the ratio of cells undergoing apoptosis, including early and late apoptosis. As shown in Fig. 8f and g, the apoptosis ratio in PC12 cells increased from 5.8% to 13.45% 24 h after the last exposure to sevoflurane. This elevation in the apoptosis ratio was mainly due to an increase in cells in late apoptosis, represented by PE^+^7-AAD^+^ cells (Fig. 8f), suggesting that neural apoptosis may be triggered by three sevoflurane exposures. The apoptosis ratio in PC12 cells infected with LV5-GLUT3 also decreased 24 h after the last exposure to sevoflurane (Fig. 8f and g). In addition, a CCK-8 assay showed that the Sev group exhibited a reduction in primary neural survival compared to the Con group, and overexpression of GLUT3 increased neuron viability to some extent 24 h after the last exposure to sevoflurane (Fig. 8h).

## Discussion

This study examined the effects of sevoflurane on cerebral glucose metabolism in young mice 24 h after the third exposure to sevoflurane and the role of GLUT3 in neural apoptosis in vitro. We found that sevoflurane may induce learning and memory dysfunction by impairing cerebral glucose metabolism in the hippocampus and temporal lobe. Multiple sevoflurane exposures in young mice also decreased GLUT3 expression and induced neuronal apoptosis. Overexpression of GLUT3 in neural cell cultures promoted glucose utilization and ameliorated neuroapoptosis. The results of this study imply that sevoflurane impairs memory consolidation by regulating glucose metabolism via affecting neural apoptosis in young mice.

Previous clinical findings have shown that children who receive multiple exposures to anaesthesia may have an increased risk of neurocognitive defects (DiMaggio et al. 2009; Wilder et al. 2009). Accumulating evidence has shown in particular that sevoflurane is toxic to neurons in the developing brain, resulting in functional deficits in neurocognition and memory ability (Liu et al. 2010; Le Freche et al. 2012; Shen et al. 2013; Zheng et al. 2013; Jia et al. 2016). Cerebral glucose metabolism and its control by the insulin signal transduction cascade are the main players in the molecular processes of memory formation and retrieval (Hoyer 2003; Zhao et al. 2004). As indicated by our analysis of PET images, glucose metabolism in the hippocampus, olfactory cortex, temporal lobe and cingulate cortex was lower in young mice than in adults. Cognitive functions are highly dependent on the hippocampus and specific cortices (Eichenbaum et al. 2007; Squire et al. 2007). Although a reduction in glucose metabolism in the hippocampus and the specific cortices is associated with cognitive dysfunction (Ouchi 1998, Silverman et al. 2001, Mosconi 2005), whether anaesthesia-induced glucose hypometabolism in the hippocampus and specific cortices has an impact on cognitive functions is unclear.

Therefore, we focused on the relationship between cerebral glucose metabolism and cognitive functions in young mice after sevoflurane anaesthesia. Sevoflurane exposure decreased glucose metabolism in the hippocampus, temporal lobe, olfactory cortex and cingulate cortex in young mice but not in adult or old mice, suggesting that the window of anaesthetic vulnerability may indeed be confined to the period of the developing brain. Young mice also exhibited learning and memory deficits in the MWM task and NOR test following sevoflurane exposure. Surprisingly, however, differences in glucose metabolism in the amygdala before and after sevoflurane anaesthesia were not apparent. The amygdala is involved in emotional and attention processes in mammals (Dicks et al. 1968; Morris et al. 1996), further supporting our results; no reduction in amygdaloid metabolism was observed in sevoflurane-exposed mice, and sevoflurane-exposed mice only exhibited a learning and memory impairment but not an increase in anxiety-like behaviour. These findings suggest that sevoflurane-induced cognitive decline in young mice may be closely associated with disruptions in glucose metabolism in the hippocampus and specific cortices.

Glucose metabolism is essential for the proper functioning of neurons and other cell types in the brain. The availability of glucose and the specific transport of glucose into the brain across the BBB and into neurons and other cell types in the brain play a key role in normal physiological function, neural activation and energy metabolism. Interestingly, among brain regions, the hippocampus and temporal lobe bear the brunt of hypometabolism (Convit 2005; Mosconi 2005), and these spots are key for processing learning and memory (Eichenbaum et al. 2007). We therefore focused on both brain regions to elucidate the mechanisms by which sevoflurane affects cerebral glucose metabolism and causes subsequent cognitive abnormalities. Our study showed that the mRNA and protein expression of GLUT3, rather than that of GLUT1 and GLUT4, were lower in the hippocampus and temporal lobe of young mice 24 h after the third exposure to sevoflurane than in those of control mice. GLUT3 is the major neuronal glucose transporter, as it is present in dendrites, axons and cell bodies that can migrate to the plasma membrane upon cellular activation to ensure increased glucose uptake and metabolism (Simpson et al. 2008; Ferreira et al. 2011). Moreover, GLUT3 expression increases predominantly during development and is expressed steadily throughout cerebral maturation (Simpson et al. 2008; Gomez et al. 2010). Thus, sevoflurane exposure at P14 mice may prevent GLUT3 expression in immature neurons at a specifically vulnerable age. The reduction in GLUT3 is a potential cause of the deficits in glucose metabolism observed after sevoflurane anaesthesia, which may induce learning and memory dysfunction in young mice.

However, our findings appear to contradict those of previous studies by Schmidt et al. (2008) and Stuart et al.(2011), as they found that downregulation of GLUT3 protein expression by loss of one allele of the GLUT3 gene (*Slc2a3*^+/−^) did not impair brain glucose uptake or affect neurological function in adult *Slc2a3*^+/−^ mice. A different mechanism may have compensated for the downregulation of GLUT3 in the brain in these mice at different ages to meet the energy demand and maintain neurological functions. The compensatory mechanism to maintain neuronal glucose metabolism in the *Slc2a3*^+/−^ adult mice may occur via the upregulation of enzymes such as hexokinase, thereby increasing the efficiency of energy production from the limited delivery of glucose to the neurons of *Slc2a3*^+/−^ adult mice, as evidenced in the muscle of GLUT4 heterozygous and homozygous null mice (Fueger et al. 2004, 2007). Although *Slc2a3*^+/−^ young mice (P21) exhibit a compensatory increase in GLUT1-mediated glucose into the brain and monocarboxylate transporter isoform 2-transported lactates into neurons, neuron-specific glucose deficiency caused by the decline in GLUT3 has a negative impact on neurodevelopment, including learning and memory impairment (Zhao et al. 2010). There is no doubt that young mice (P14) in our study were unable to establish a compensatory mechanism in response to acute decreases in GLUT3 and glucose hypometabolism caused by sevoflurane anaesthesia, resulting in learning and memory impairment.

Normally, GLUT3 functions at the cell surface and is recycled between the plasma membrane and intracellular synaptic-like vesicles to maintain cellular glucose homeostasis (Thoidis et al. 1999). In the present study, immunofluorescence and WB analysis also demonstrated that sevoflurane significantly diminished total GLUT3 expression in cultured neurons 24 h after the last exposure to sevoflurane. Most importantly, upon flow cytometry analysis of PC12 cells labelled with the peptide in the first extracellular loop of GLUT3 and G6P expression in primary neurons, sevoflurane was shown to reduce the translocation of GLUT3 to the plasma membrane, resulting in significantly decreased levels of intracellular glucose. Furthermore, neuronal cells were transfected with lentivirus overexpressing GLUT3 or empty vector, and GLUT3 overexpression ameliorated the sevoflurane-induced decline in GLUT3 translocation and glucose metabolism. These results imply that sevoflurane caused glucose hypometabolism in neurons of the hippocampus and temporal lobe by reducing the total expression and surface expression of GLUT3.

The effects of general anaesthetic on foetal and neonatal *N*-methyl-d-aspartic acid (NMDA) glutamate receptors or gamma-amino-butyric-acid type A (GABA_A_) receptors activate the caspase pathway, leading to an apoptotic cascade (Satomoto et al. 2009; Kodama et al. 2011; Creeley et al. 2014; Ye et al. 2018). In addition to inducing neuroapoptosis via the NMDA and GABA_A_ receptors, anaesthesia-induced neuroapoptosis in the developing brain may occur through glucose metabolism pathways. Our results demonstrated that sevoflurane caused glucose hypometabolism and apoptosis in the hippocampus and temporal lobe 24 h after the third exposure to sevoflurane. As expected, GLUT3 overexpression in neurons alleviated sevoflurane-induced glucose hypometabolism and neuroapoptosis, consistent with previous studies showing that a decline in glucose transport induces cellular apoptosis through activation of the caspase pathway (Richter et al. 1996; Mueckler 2000).

Notably, it is unknown whether overexpression of GLUT3 in specific brain regions can restore sevoflurane-induced hypometabolism and neuroapoptosis and reduce sevoflurane-induced cognitive deficiency in the developing brain. Further investigations are warranted to test this hypothesis.

In conclusion, we demonstrated that GLUT3, the predominant neuronal glucose transporter, plays a key role in sevoflurane-induced glucose hypometabolism and neuroapoptosis. These results imply that sevoflurane causes cerebral glucose hypometabolism by reducing GLUT3 expression, which induces neuronal apoptosis, finally leading to cognitive impairment in young mice.
